# Hospital and Institutional News

**Published:** 1914-01-24

**Authors:** 


### HOSPITAL AND INSTITUTIONAL NEWS.

### NEW CLINICAL LABORATORIES AT WOLVERHAMPTON.

The Wolverhampton and Staffordshire General Hospital has hitherto been dependent on Birmingham and London for bacteriological and clinico-pathological aids to diagnosis and treatment. Last week, however, Sir Clifford Allbutt opened new laboratories which will enable all this necessary work to be done on the hospital premises, an improvement which was insistently necessary. The new laboratories appear first-class in design and equipment, and will suffice, not only for the needs of the hospital itself, but also for those of the surgeons and practitioners of the town and district, and also for the veterinary surgeons. They have cost £1,400 in all, and will be in charge of Dr. W. Boyd, who was appointed pathologist at this institution in June last. Sir Clifford Allbutt, in his opening speech, gave some interesting details of the history of the hospital, as well as a stimulating account of the inter-relations of pathology and medicine. In 1845 Mr. George Briscoe broke his leg, and while convalescing the idea occurred to him of founding an institution for those who could not afford to pay for skilled treatment in illness. In 1849 the hospital was opened; a children's ward was added in 1862, and an isolation block in 1872, in which year also the out-patients' department was extended. In 1877 the Bell Library was built; in 1894 the *post-mortem* room was built; and in 1896 the operating theatre was modernised. A nurses' home in 1908 and a King Edward Memorial Wing in 1912 complete the tale of progress from small beginnings. The plan to instal a laboratory of this kind was reported in The Hospital last May.

### THE MEDICAL STAFF OF THE LEICESTER POOR-LAW INFIRMARY.

The medical arrangements at the North Evington Poor-Law Infirmary, Leicester, have been under discussion by the local Board of Guardians for some months. At present the infirmary has a staff composed of a visiting medical officer and two assistant resident medical officers. Some months ago it was suggested that this staff should be replaced by a resident medical superintendent at a salary of £500 per annum, one resident assistant medical officer at £150, and a visiting surgeon and physician at £100 each—the first two officers to be provided with board and residence in addition to their salaries. At a recent meeting of the Board of Guardians the matter was carried a step further. The appointment of a medical superintendent at a salary of £400 rising to £450 in two years, with emoluments of the value of £80, was discussed and approved of—the question whether the salary should include extra fees and some other details being left to a committee for discussion and report. It was further suggested that a visiting surgeon and physician should be appointed at a salary of 52 guineas each, but after discussion it was finally decided that both posts should be honorary ones. It may be safely prophesied with regard to the two visiting appointments that this attempt to exploit the charitable instincts of the medical profession will fail. About two years ago the Brighton Guardians tried to obtain a visiting surgeon for the local workhouse infirmary for an honorarium of 50 guineas per annum.

### THE PROPER SALARY FOR AN INFIRMARY SUPERINTENDENT.

The Brighton division of the British Medical Association decided that fifty guineas was inadequate, with the result that no suitable applications for the post were received and the salary had to be raised to 100 guineas. This action may be recommended to the Leicester division of the British Medical Association for their adoption. As for the post of medical superintendent one optimistic Guardian was of the opinion that the salary offered was so munificent that they would get a man who would not need to consult anyone. In the face of such high hopes we feel almost sorry to have to say that the salary appears insufficient to attract any medical man with the necessary experience and qualifications. The North Evington Infirmary contains 520 beds, receives about 1,800 patients annually, and is a training school for nurses. The salary for an efficient medical superintendent of such an infirmary should be at least £500 rising to £600, exclusive of extra fees and emoluments. Boards of Guardians certainly lose sight of the fact that medical superintendentships are the prizes of the Poor-Law Medical Service, and, unless the terms of appointment assigned to them are attractive, the service will not attract well-qualified and hard-working men to the junior posts. The result is the whole work suffers in efficiency and economy alike.

### THE PAYMENT OF HOSPITAL CHAPLAINS.

A representative of Labour at Lincoln raised the question at a meeting of governors of Lincoln County Hospital whether or not the chaplain should be paid. The people the speaker represented put forward their case on the thinly-disguised ground that the chaplain's services were worth nothing. We are glad to note that the board took an opposite view, and Mr. J. Dutton's suggestion that the duties should be done voluntarily—the chaplain's salary is £80 a year at present—found no seconder. Mr. C. C. Sibthorp, who was in the chair, pointed out that the chaplain attended every day, and that it was essential to have one man to rely on in emergencies, while the alternative suggestion of having half-a-dozen unpaid chaplains was shown by Dr. Carline and Mr. Sibthorp to be administratively impossible. Now the fundamental objection that the services of hospital chaplains are of no value may be dismissed at once. It is purely theoretical in origin, and can be contradicted practically by every man and woman who has had any active part in hospital life, and if by many patients no one's services are more valued, few have more exacting work to perform. The administrative side of the question is also worth notice. Although the voluntary hospital system relies to a vast extent upon honorary and unpaid work, the work which is undertaken with no view to the value of the experience gained, as in the case of the honorary staff, which is at the same time routine work day by day, and for which some one must be responsible in an emergency can only be unpaid in the rare cases, where a person is able and willing to place his whole time at the service of the institution. Most of the unpaid work is not of this regular routine nature, involving rather a visit once a week than once a day, and as a rule no immediate likelihood of responsibility in an emergency. The chaplain's work, on the other hand, involves both routine and prompt response to a call at any time. These, apart from the high value of the work itself, make the payment of hospital chaplains a desirable feature in any up-to-date administration.

### THE CONTROL OF RADIUM IN AMERICA.

A Bill for the conservation of all radium ore deposits by the Government has been introduced at Washington. The therapeutic value of radium and the extent of its export to Europe have simultaneously impressed public opinion, and the House Committee on Mines is starting an inquiry into the therapeutic value of radium and the disposition of the ore throughout the States. At present, says the *Times* correspondent, only two companies appear to be engaged in the manufacture of radium, one of which is reported to have large contracts for its export. As the same authority points out, the truth is that Europe has been ready to outbid the States for this product, and legislation would find a difficulty in the fact that an important fraction of the land which yields radium is already in the hands of private persons.

### EARLY MEDICAL LITERATURE.

Sir William Osler, Regius Professor of Medicine at Oxford, delivered, as President of the Bibliographical Society, an address last Monday on "The Earliest Printed Medical Books." He pointed out that documents called Calendars were the earliest medical compilations, and that their popularity was bound up with the prevailing belief that the phases of the moon and the conjunctions of the planets powerfully affected the therapeutic value of drugs and surgery. Arabian influence had left a permanent mark on pharmacy, and the British Pharmacopœia owed many drugs—for instance, senna, mercury, alcohol, essences, and extracts—to Arab doctors. In 1470 a volume called "The Agregator" was published, being in fact a dictionary of treatment, of the kind, added the lecturer, still called for by a certain type of practitioner who dealt in "penny-in-the-slot" treatments. We are glad that the persistence of old methods to our own times was thus insisted on, for the study of the past in such regions as folklore, medicine, astrology, and politics, for instance, loses most of its value unless it is shown to be as alive as the dictionary referred to.

### THE CENTRAL POOR-LAW CONFERENCE, 1914.

This Conference will be held on February 17 and 18 in the Council Chamber of the Guildhall, London. Field-Marshal Lord Methuen will be the president and will give an address. On the first day a paper will be read by Mr. Curtis, the clerk of the Birmingham Guardians, on "The Poor Law and the Care and Training of Mentally Defective Persons." On the second day the morning session will be devoted to a paper by Mr. Leach, clerk of the Rochdale Guardians, on "The Overlapping of Old-age Pensions, National Insurance, and the Poor Law," and the afternoon to papers on "Housing as Affecting Pauperism." Miss Cochrane, a member of the St. Neots Board of Guardians, will deal with the latter subject as regards rural districts, and Mr. Nettlefold, chairman of Harborne Tenants, Limited, as regards urban districts. All the papers will be followed by discussion. The subjects selected are eminently practical and important ones, and though the first only is of immediate importance to institutional workers, the reputation of the readers of the papers promises valuable and stimulating contributions to the problems which will be discussed.

### THE USE OF RADIUM IN AUSTRALIA.

In the recent Report of the Alfred Hospital, Melbourne, for the year ended June 30, 1913, the following paragraph appeared: "In December last the Premier generously offered to supply to this hospital £1,500 worth of radium for £500. Much as the managers would have liked to accept such a tempting offer, lack of funds prevented their doing so, while the medical staff, who were consulted in the matter, though fully recognising the desirability of possessing such a supply of radium for experimental purposes, agreed that there were other directions in which an expenditure of £500 was more urgently needed." Shortly after its publication, a professional bookmaker, whose benevolence is widely recognised in the State of Victoria, forwarded £250 of the required amount. To this succeeded £200 and the balance was speedily subscribed, thus enabling the hospital board to accept the Government's offer. A few weeks later a sum of £1,000 was allocated to the same institution from the "Walter and Eliza Hall" Trust, wherewith to build and equip a pavilion for the $x$-ray department. As the Alfred Hospital already possesses a fairly good electrical and $x$-ray plant, together with a modicum of radium, the acquisition of this extra money, if judiciously expended, should go far towards placing the hospital in the front rank as regards these special branches of medical work.

### LISTER MEMORIAL TABLET AT KING'S COLLEGE.

Lord Rayleigh unveiled a memorial tablet to the memory of Lord Lister at King's College last week. An impressive ceremony in the Chapel preceded the unveiling, and Lord Rayleigh and the Vice-Chancellor, Dr. W. P. Herringham, delivered addresses. In the course of his dignified and touching tribute Lord Rayleigh recalled that he had served under Lister for a time at the Royal Society, and that then and afterwards he was struck by the importance of his work and the extreme modesty with which Lister regarded it. All who have read Dr. Wrench's recent "Life" will appreciate Lord Rayleigh's statement that "he was shy of publishing his early operations, fearing that it might lead others to similar attempts without the precautions which he deemed necessary." The tablet has been placed in the corridor outside the Chapel, and is thus inscribed: "In affectionate and respectful memory of Joseph Baron Lister, F.R.S., O.M., Professor of Clinical Surgery in King's College from 1877-1892, and for many years consulting surgeon to the King's College Hospital, Member of the Council and Life Governor of the College, this tablet is erected. His name will be handed down to posterity as the founder of anti-septic surgery, one of the greatest discoveries in history and a source of inestimable benefit to mankind." The lesson drawn for the daily life of the modern surgeon by the speaker was Lister's application of the rule "do as you would be done by" when considering the question of an operation. The more elderly note with disquiet signs sometimes of the surgeon developing into two classes, the conservative and the "bold." Lister was always the apostle of caution and care. But the question remains: How is the larger memorial scheme advancing?

### A RATE-SUPPORTED HOUSE SURGEON.

The Barry District Council, whose accident and surgical hospital is described as the only institution of its kind in the country wholly supported by local rates, has decided to appoint a house surgeon at a salary of £150, with emoluments, rising to £200 a year. The Chairman, Councillor J. T. Davies, in opposing an alternative proposal to appoint a resident surgeon at £450, with an assistant at £150, urged that the co-operation of medical men in the town had caused very successful work to be done at the hospital, and this view was accepted by the Council, which voted in favour of a house surgeon at the above salary. The hospital, which was taken over by the Urban District Council from the local District Nursing Association in 1900, is only for accidents and was founded in 1895. The committee of management is appointed partly by the Council and partly by the local medical men, of whom eight surgeons and eight physicians form the staff. There are only twenty-six beds, and the institution is an interesting and isolated example of an emergency hospital in this country run on rate-supported lines.

### A GREAT PRIVATE MENTAL HOSPITAL.

Only a few weeks ago, October 25 last, we remarked on the curious tendency to specialise in lunacy which crops out in generation after generation of certain families well known among alienists. Another instance of this tendency is brought to mind by the death on January 17 of Dr. Alexander Newington, joint proprietor with his cousin, Dr. Hayes Newington, of the private asylum at Ticehurst, Sussex. This institution was founded early in the nineteenth century by the grandfather of the present proprietors, and at least two sons and three grandsons of the original Dr. Newington have had a share in its management. At the present time, if not actually the largest private mental hospital in this country, it is certainly one of the most sumptuous and celebrated. Probably no similar institution could lay claim to patients from so many well-known families; and its reputation for care and efficient treatment has always been of the highest. The late Dr. Alexander Newington had other interests besides the insane: he was at one time an Indian planter, had been well known as a rifle shot, and was a most expert motorist, having been amongst the first to take up this hobby before the end of the last century. Dr. Newington was a St. Thomas's Hospital man; he qualified M.R.C.S., L.R.C.P. in 1874 and M.B. (Cantab.) in 1875.

### SUNDAY AND SATURDAY FUND DECREASES.

It is instructive to place side by side this year the totals realised by the Metropolitan Hospital Sunday and Saturday Funds, for both show unfortunately marked decreases. The collections for Hospital Sunday in London for 1913, analysed as usual by the *National Church* to explain the amounts respectively contributed by the various denominations, totalled £28,410, the net falling off in the collections being some £3,000, now that donations amounting to £4,000 are no longer included in the collection at St. Paul's Cathedral. The total receipts of the Hospital Saturday Fund are given as £40,272, a decrease of practically £5,000 on the total received for 1912. There is this reassuring fact, however, to be noted, that during the last quarter of the year the income not merely ceased to shrink at the rate of £100 per week, but actually was an advance upon the income for the corresponding period twelve months before. Mr. A. W. Davis, the secretary, points out that considering the perplexity caused to Saturday Fund subscribers the amount of the decrease is relatively small, and the honorary collectors and local committees deserve credit for the way in which the arduous task that they had at the beginning of the year was surmounted by their enthusiasm. The falling off in the Sunday Fund receipts is less satisfactorily explained.

### HOSPITAL CLERK BURNT TO DEATH.

An inquest was held recently by Dr. Waldo, the Coroner, at Southwark, on the body of Miss Doris Marie Drake, a clerk at Guy's Hospital, whose death occurred from her dress catching on fire in the out-patients' visitors' office. Miss Drake was the step-daughter of Mr. Alfred Hooper, who is assistant secretary to the Cancer Hospital. Evidence was given by Miss Hilda M. Price, another clerk at Guy's, who said that Miss Drake, while dictating to her, had been standing with her back to an open fire, to which there was no fender.

Suddenly she exclaimed, "My apron is alight!" and ran into the out-patient hall, while Miss Price attempted to smother the flames in an out-patient's cloak, calling, meanwhile, for assistance. Dr. Ozanne said that death was due to shock, and the jury, in returning a verdict of "Accidental death," added a rider that all fireplaces in the hospital to which women had access should be guarded. Why they did not simply state all fireplaces, without this curious sex qualification, is not clear. The Coroner made an interesting attempt to get evidence of the class of shoes worn by the deceased. He pointed out that shoes of the smartest kind now worn were often furnished with celluloid buckles, and the heels frequently covered with sheet celluloid or with a less dangerous varnish made from celluloid and spirit. The fumes given off by this material when warmed were liable suddenly to flame and set fire to clothing. He added that in this case the deceased was standing only a foot and a half away from the fire, and that ladies as a rule knew nothing of their danger. We trust that this sad accident will lead not only Guy's, but other hospitals, to overlook their precautions, or lack of precautions, in this respect. A hospital should be the last place where such necessary and obvious precautions are neglected.

### REMARKABLE GIFT TO ASHTON INFIRMARY.

Nearly two years ago, on March 16, 1909, we recorded the gift of a cot by Mr. and Mrs. Edward Belfield to the Ashton District Infirmary. On that occasion we remarked on the astonishing thrift and persistence which had led the donors, who have spent most of their lives as cotton spinners, to save so large an amount as is required to endow a cot at a hospital. The idea was to celebrate their golden wedding, which was to have occurred this year, but Mr. Belfield, rightly as we have to record with regret, feared that he would not live to enjoy this anniversary. Mrs. Belfield, however, is carrying out the letter of her late husband's earlier intention, and last week sent £1,000 to the hospital to endow a cot in memory of her father and mother. Thus, in addition to the "Jubilee" cot endowed two years ago, a second is to be endowed at the institution through the generosity of these remarkable examples of thrift. The name of Belfield will have an honoured, and indeed unique, place in the history of Ashton Infirmary.

### HIGHER SALARIES FOR LONDON INFIRMARY STAFFS.

At a recent meeting of the Fulham Board of Guardians it was decided, subject to the approval of the Local Government Board, to raise the salary of the senior assistant medical officer from £175 to £200 by two annual increments of £12 10s.; that of the second assistant medical officer from £110 to £125, at the end of one year to £140, and then by two annual increments of £10 to £160; and that of the junior medical officer from £100 to £110. A return of the salaries at present paid to the assistant medical officers in the twenty-six London infirmaries shows that the difficulty experienced in obtaining suitable men for these posts has led to a general increase in these salaries in the last two years. Assuming the assent of the Local Government Board to the scale proposed at Fulham, eleven Boards of Guardians—viz., Bermondsey, Camberwell, the City of Westminster, Fulham, Greenwich, Hackney, Islington, Lambeth, Mile End, the Poplar and Stepney Sick Asylum district, and Wandsworth now pay their senior assistants a salary of £200 and upwards; six—viz., Bethnal Green, the City of London, St. Marylebone, Shoreditch, Southwark, and Whitechapel pay from £175 to £200; and ten—viz., Chelsea, Hammersmith, Holborn, Kensington, Lewisham, Paddington, St. Giles, St. Pancras, and Woolwich pay less than £175. A comparison with a similar return obtained in January 1912 shows the advance that has been made. At that time, only five Boards of Guardians paid a maximum salary of £200 and upwards to their senior assistants, only two paid between £175 and £200, and nineteen paid less than £175. A further study of the two returns shows that twenty out of the twenty-six Boards have increased the maximum salaries of their senior assistant medical officers in this period. Similar increases have also been made in the case of the junior assistant medical officers. There is no doubt that those Boards which have not already done so will soon be compelled to increase the salaries they offer, owing to inability to fill vacancies under the old scales.

### MARRIAGE AND THE WASSERMANN REACTION.

Several of the United States have enacted more or less stringent laws to prevent the marriage of diseased or degenerate persons by requiring medical certificates of fitness for parentage. One State, however, has gone further still, and has, incidentally, run itself up against a brick wall. It has not only passed eugenic legislation on the lines just mentioned, but has ordained also that the medical examination shall include a Wassermann reaction, and that the statutory fee for the whole examination shall be three dollars. The doctors decline to accept this fee, which would leave them heavily out of pocket over each case; and a deadlock has ensued, which is preventing the marriages of several couples desirous of contracting matrimony. Legislation of this kind will have the natural result of bringing eugenic ideals into disfavour and disrepute, a most unfortunate result of trying to go ahead too fast. In this connection it is of interest to note that McDonagh is now doubtful whether the present interpretations of the Wassermann reaction are correct; he claims to be able to make the reaction positive or negative at will. If this conclusion is confirmed by other workers, the absurdity of the American legislation on this point is rendered even greater, and its results even more unjust.

### HOSPITALS AND THE WORKMEN'S COMPENSATION ACT.

Arising out of a charge that a patient's death had been hastened by removal from the Hereford General Hospital, Sir Archer Croft has made some interesting proposals. He first urged that, where a county case came into the hospital and developed infectious disease and had to be sent to the nearest isolation hospital, it was the duty of the District Councils either to pay the fees that were due to the isolation hospital, or to pay the cost of isolating the patient at home. So far, in spite of his attempts, the District Councils had given him no satisfaction. Another proposal with reference to income was that the Income-Tax Commissioners should be asked to allow the hospital to charge for cases which were received under the Workmen's Compensation Act, so as to recover the out-of-pocket expenses. Sir Archer Croft said that he supposed at least 75 per cent. of accidents to men which came to the hospital were really insured cases under this Act. In fact, employers had often written to the secretary, Mr. Baxter, asking for "a bill" for the medical attendance of workmen who had been treated for accidents. The Secretary had to decline, for if he did not the hospital was liable to pay income tax on all its revenue on the ground that it was trading for profit. They were probably losing hundreds of pounds a year in not being able to receive recompense for this class of service. A deputation to approach the Income-Tax Commissioners, consisting of Sir Geoffrey Cornwall, Bart., chairman, Sir Archer Croft, and Mr. W. J. Humphrys, was appointed. If the employers are as ready to pay for medical attendance to their workmen who have met with accidents and whom they have insured under the Workmen's Compensation Act, as was stated, surely there should be little difficulty in answering their request for "a bill" with a letter explaining the hospital's position and at the same time concluding with a reminder that subscriptions were for this reason all the more welcome. Why not?

### PROBLEMS OF DISCIPLINE AT WINSLEY SANATORIUM.

Some weeks ago Dr. Bathe undertook the duties of resident medical officer at Winsley Sanatorium, and, as is not altogether surprising to those who remember from our columns the difficulties which have beset its administration in the past, he found what he has described as a very undesirable lack of discipline among the patients. Thereupon certain new rules were introduced, which were the subject of some discussion at a meeting of the house committee last week. On Mr. Frank Moore, the chairman, inviting an explanation of the changes made, Dr. Bathe referred to the indiscipline that he had encountered, and said that he had consulted with the matron and other officers with the result that certain new rules had been drawn up. His aim had been to secure discipline without interference with the reasonable liberty of the patients. Opinions were expressed supporting the contention that discipline had been unsatisfactory, and in the end the committee approved what Dr. Bathe has done. It will be remembered that last year we were compelled to criticise the policy which gave the resident medical officer at this sanatorium the responsibilities without the authority of a medical superintendent, and we hope that the difficulties which directly resulted from this disastrous confusion of principle are sufficiently present to the authorities to make them unwilling ever again to pursue that divided course.

### A PRIZE FOR MEDICO-LEGAL WORK.

At first sight the connection between the institutional world and the Swiney Prize for jurisprudence is not obvious, but it exists from the fact that the prize itself was the foundation of an eccentric medical man, who appointed the Fellows of the Royal College of Physicians to be among the adjudicators. The prize which consists of £100, contained in a silver cup of the same value, and is awarded every fifth anniversary of the founder's death to the author of the best published work on jurisprudence, has been adjudged this year to Mr. John W. Salmond, Solicitor-General to New Zealand, for his "Jurisprudence." There have been thirteen previous awards, the recipients including such famous names as Sir Henry Maine for his "Ancient Law," T. E. Holland for his "Elements of Jurisprudence," and Sir F. Pollock and Professor Maitland for their "History of English Law before Edward I." Among these three, it will be noticed, no outstanding name of a modern medical jurist is included. Perhaps the fascinating study of medical jurisprudence will produce its modern crop of jurists only when every Coroner is compelled to be a qualified practitioner. Presumably this department of law must have been the chief interest of Dr. George Swiney, and one which he wished more particularly to foster.

### THE LONDON HOSPITAL'S APPEAL.

We are glad to learn that the quinquennial appeal on behalf of the London Hospital has been successful, and that the sum of £100,000 asked for has been raised. It will be remembered that a gift of £15,000 from the Commercial Union Assurance Company, as trustees of the late Sir William Dunn, was conditional upon a similar sum being raised by January 31. Fortunately, this condition has been fulfilled, and so the institution, with its most urgent needs satisfied for the moment, may turn to its annual labour of raising £113,000 for its ordinary work. The success of the appeal, and of the policy which underlies its regular issue, is thus again shown, and should encourage the authorities to pursue the new developments which they have never been slow to undertake. Prominent among these is the new ward to be devoted to salvarsan treatment, for which funds have still to be found; and we hope that this policy of initiative, alike in the way of appeal and in the progress of the hospital's work, will not be lost on any other institutions who are lamenting the difficulty of raising money nowadays.

### NORTH STAFFORDSHIRE INFIRMARY PROBLEMS.

Some remarkable speeches were made at the annual meeting of the governors of North Staffordshire Infirmary last week, when an urgent demand was made for more annual subscribers, and, particularly on the part of Colonel Heath, for an up-to-date operating theatre. The gratifying fact is that the income of the institution has increased during the past year by over £1,200, while what are known as the establishment subscriptions have reached the fine total of very nearly £8,000. With it all, however, there was a loss on the year's working of some £2,500, and when this is added to the deficit of £4,000 with which the year began, the financial problem becomes apparent. The regular income must be substantially raised, and seeing the fine example set by the establishment subscribers and by the Saturday Fund, it is only fair that the middle and richer classes should supplement the strenuous efforts of those who can subscribe only small weekly sums. These sums, and the interest and enthusiasm which they indicate are invaluable—in fact, the best testimony to the hospital's position in the county, but no voluntary hospital must depend on this class of subscriber alone or mainly. The annual subscribers of a guinea and upwards and the donations must increase, and will do so if the hearts of this section of the local public are as warm as Mr. A. J. Johnson, the retiring president, believes them to be. There is much to indicate, however, that this class of giver in North Staffordshire has not been doing his duty of late. For instance, in urging that the building committee must undertake a new operating theatre at the earliest moment, Colonel Heath declared the existing accommodation for operating to be "worse than that of any first-class hospital in the United Kingdom." Now such a state of things is precisely what donors and annual subscribers, as a rule, combine to prevent. In this case they have an opportunity to retrieve their reputation. We hope that they will do so, and thus make Mr. F. J. Harrison's year of presidency remarkable in the best sense.

### BARBADOS GENERAL HOSPITAL.

THE Barbados General Hospital, situated in Bridgetown, which though not, strictly speaking, a Government institution, is maintained by a legislative grant, and governed by a court of directors of eighteen members, of whom all, save two life members, must either be Government officers, sitting *ex officio*, or members of the Legislature, accommodated according to the last returns a daily average of 200. During the past year considerable extensions to the buildings were carried out, greatly increasing the accommodation hitherto provided. The new buildings were formally opened by Princess Marie Louise of Schleswig-Holstein on April 28, 1913, and Her Highness accepted—as a memento of the occasion—a cup recording, in Latin, the inauguration of the extension.

### BIRTHDAYS.

MR. H. JOHNSON, the able secretary to the Leicester Royal Infirmary, has made a most excellent suggestion which we hope will commend itself to hospitals all over the country. It is to mark the occasion of the birthdays of the older greater benefactors and workers at each institution, by sending to THE HOSPITAL a short account of their work or the work of one or more of them from time to time. Such birthday records, if a little care is taken in their preparation, should help the hospitals and popularise them too.

### THE INSURANCE ACT AND PROPRIETARY MEDICINES.

THE question of the prescribing of proprietary medicines continues to engage the attention of Insurance Committees in various parts of the country, and most acutely in those districts in which the drug funds are hardly likely to be sufficient to pay the chemists' bills in full. In some of the places where there is a large deficiency, it is said that this is to some extent due to extravagant prescribing, but in other places where panel doctors have had an equally free hand, there is not only enough to pay the chemists in full, but the doctors will get at least a portion of the "floating sixpence." The excess is, therefore, not wholly due to the prescribing of proprietary medicines, and obviously the question of excessive sickness must be taken into account. The general tendency of the policy of the Insurance Committees is to discourage, if not to prohibit, the prescribing of proprietary medicines of secret composition, but to allow the chemists to dispense prescriptions containing proprietary medicines, the composition of which is known. This policy is perhaps the wisest under the circumstances, and little harm can be done by discouraging practitioners from prescribing compounds of which they do not know the composition.

### THIS WEEK'S DRUG MARKET.

THERE is still plenty of room for improvement in the Drug market, and, although business is brighter than it was a month or so ago, it is still on the quiet side. Much interest is being taken in several articles, and there is a fair demand for drugs which are largely prescribed during the cold season. Definite news which would enable an opinion to be formed of the prospects of the cod fishing in Norway is still lacking, but it appears that stormy weather is prevailing in the fishing grounds, which prevents much progress being made at present; in the meantime, buyers of cod-liver oil are still holding off, and no business of importance is likely to be done until more definite information is available. Considerable business has been done in bromides, and the opinion is held in some quarters that an advance in prices is imminent, but this is by no means certain. Quinine also continues to be in good demand, but makers' prices are unchanged; an advance in price sooner or later seems inevitable, but the general view is that it will be delayed for a short time. Menthol is quiet, but there has been no further decline in value, the tendency being in the other direction. Opium is quiet and the position practically unchanged. The price of castor oil is somewhat lower. Thymol is dearer. Citric acid has a downward tendency. American oil of peppermint tends upwards in value.

